# Supplementary material for: Landscape of Well-Coordinated Fracture Healing in a Mouse Model Using Molecular and Cellular Analysis
Source: Int J Mol Sci. 2023 Feb 10;24(4):3569. doi: 10.3390/ijms24043569 (PMC9964763; doi:10.3390/ijms24043569)
Supplement: Supplementary file 1 [file ijms-24-03569-s001.zip › ijms-2155586-supplementary.pdf]

Landscape of well-coordinated fracture healing in a mouse model using molecular and cellular analysis

Deeksha Malhan[1,2#], Katharina Schmidt-Bleek[3,4#], Georg N. Duda[3,4#], Thaqif El Khassawna[1#*]

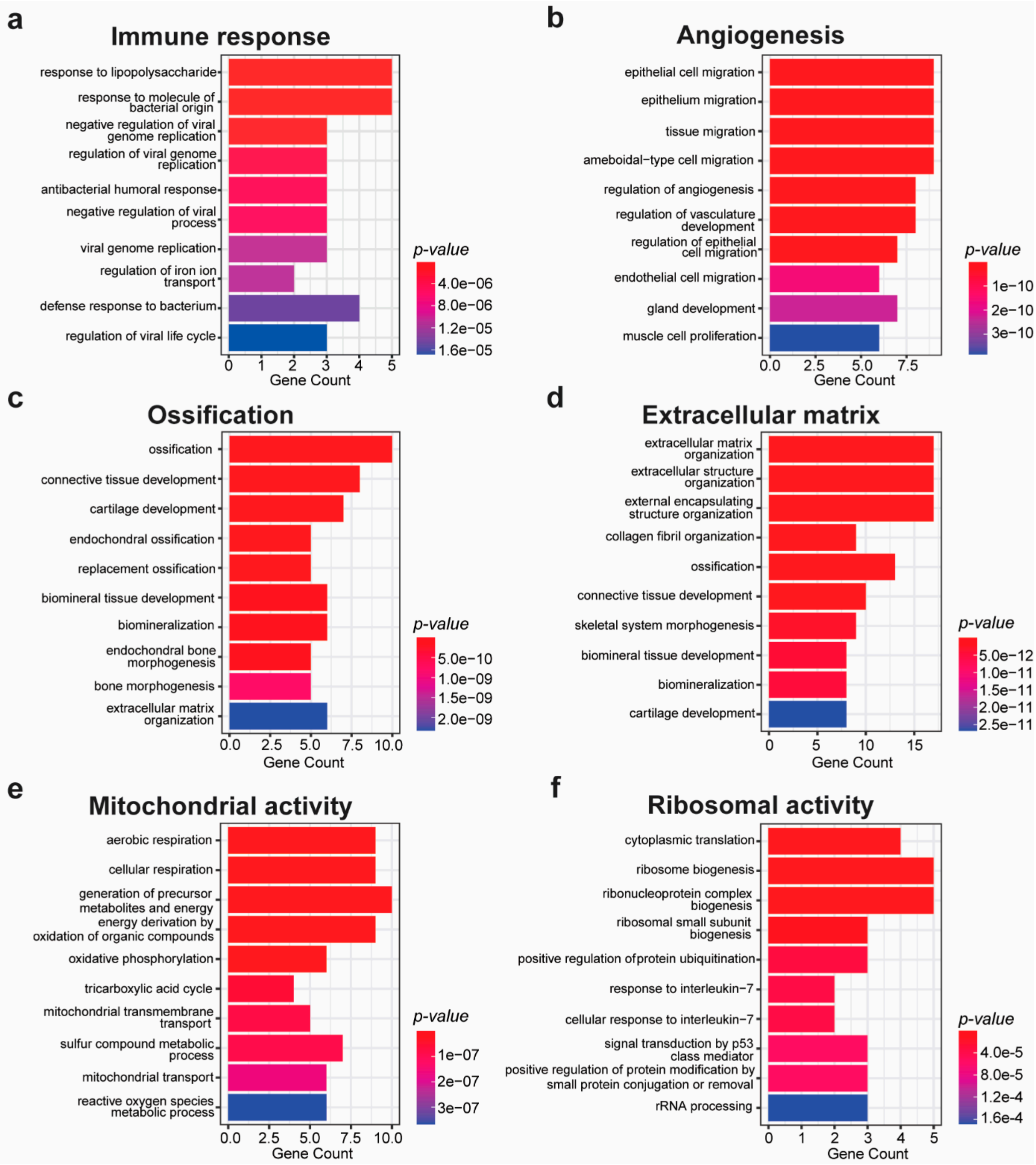

Figure S1*:* Functional enrichment analysis depicts the top 10 enriched biological processes associated with differentially expressed genes involved in (a) Immune response, (b) Angiogenesis, (c) Ossification, (d) Extracellular matrix organization, (e) Mitochondrial activity, and (f) Ribosomal activity.

Table S1*:* List of differentially expressed related to the angiogenesis process along with their FC and P-value (white background: downregulated and grey background: upregulated)

Gene	D3	D7	D10	D14	D21	D28
Vegfa	-2.313<br>6.46E-05	-2.236<br>1.91E-04				
Ptn	-2.374<br>2.17E-05	-2.2878<br>4.64E-05			-2.179<br>6.65E-05	-2.292<br>1.34E-05
Dcn	-2.35442<br>3.14E-04	-2.373<br>3.10E-04				
Aqp1	-2.39845<br>5.61E-06	-2.32452<br>2.21E-05				
Gpx1	-2.95<br>7.9E-06	-3.164<br>8.9E-06				
Mmp2	-2.094<br>1.07E-05	-2.262<br>5.92E-05				
Mapk14	-2.713<br>2.16E-05	-2.759<br>1.43E-04				
Serpinf1	2.386<br>8.1E-07	2.196<br>2.65E-05				
Mmp9			2.3381<br>1.30E-05	2.34614<br>1.26E-05	2.19513<br>5.35E-06	
Tgfbr1				2.1474<br>3.79E-05		
Tgfbr2			2.042<br>7.46E-05			
Sparc			2.9793<br>1.85E-06			

Table S2: List of differentially expressed related to the ossification process along with their FC and P-value (white background: downregulated and gray background: upregulated)

Gene	D3	D7	D10	D14	D21	D28
Ubb	-3.837<br>5.82E-08	-3.823<br>3.13E-06				
Mef2c			-2.582<br>8.99E-05	-3.08<br>1.67E-04		
Col1a1			3.073<br>7.99E-06	2.745<br>1.16E-05	2.038<br>3.86E-05	
Col10a1			3.1519<br>2.21E-07			
Col2a1			5.08<br>9.26E-08	2.982<br>8.81E-06		
Mmp9			2.3381<br>1.30E-05	2.34614<br>1.26E-05	2.19513<br>5.35E-06	
Mmp13	2.05<br>4.11E-06	2.166<br>3.73E-06	2.235<br>5.46E-05	2.36<br>3.83E-06	2.291<br>1.90E-06	
Timp1	2.2396<br>2.55E-05	2.2505<br>1.02E-05			2.2906<br>4.71E-06	
Ctsk			2.6148<br>3.75E-06	2.54762<br>5.01E-06	2.26895<br>2.20E-05	
Sp7	2.867<br>4.85E-07	2.833<br>9.27E-05		2.107<br>3.04E-06		
Mgp			2.119<br>1.62E-06			
Spp1			2.434<br>3.51E-04	2.313<br>5.41E-04		
Ibsp	3.345<br>9.05E-06	3.379<br>8.26E-05	2.821<br>3.40E-05	3.008<br>3.00E-04	2.956<br>2.51E-05	

Table S3: List of differentially expressed related to the ECM regulation along with their FC and P-value (white background: downregulated and gray background: upregulated)

Gene	D3	D7	D10	D14	D21	D28
Mmp2	-2.094<br>1.07E-05	-2.262<br>5.92E-05				
Ogn	-2.401<br>3.72E-05	-2.078<br>2.20E-03				
Dcn	-2.354<br>3.14E-04	-2.373<br>3.10E-04				
Lum	-3.642<br>2.22E-05	-3.55<br>1.7E-03				
Col12a1		-2.052<br>4.80E-04				
Ptn	-2.374<br>2.17E-05	-2.287<br>4.64E-05			-2.179<br>6.65E-05	-2.292<br>1.34E-05
Mmp13	2.05<br>4.11E-06	2.166<br>3.73E-06	2.235<br>5.46E-05	2.36<br>3.83E-06	2.291<br>1.90E-06	
Mmp9			2.338<br>1.30E-05	2.346<br>1.26E-05	2.195<br>5.35E-06	
Ibsp	3.345<br>9.05E-06	3.379<br>8.26E-06	2.821<br>3.40E-05	3.008<br>3.00E-04	2.956<br>2.51E-05	
Timp1	2.239<br>2.55E-05	2.25<br>1.02E-05			2.29<br>4.71E-06	
Tgfbr1				2.147<br>3.79E-05		
Tgfbr2			2.042<br>7.46E-05			
Sh3pxd2b	2.1583<br>3.02E-06	2.0547<br>6.34E-06				
Efemp2	2.04<br>2.70E-06	2.1007<br>9.64E-06				
Mgp			2.119<br>1.62E-06			

Tnc			2.811<br>7.41E-08	2.585<br>1.67E-06		
Acan			3.268<br>1.54E-07			
Crtap			2.166<br>8.87E-10			
Spp1			2.434<br>3.51E-04	2.313<br>5.41E-04		
Cpz			2.404<br>3.27E-06	2.096<br>4.02E-05		
Col24a1		2.126<br>2.9E-03				
Col6a1			2.019<br>3.81E-06			
Col1a1			3.073<br>7.99E-06	2.745<br>1.16E-05	2.038<br>3.86E-05	
Col1a2	2.322<br>1.81E-04	3.109<br>1.23E-04	2.63<br>9.32E-05	2.88<br>5.28E-05		
Col10a1			3.151<br>2.21E-07			
Col9a2			2.903<br>1.36E-05	2.148<br>2.14E-04		
Col2a1			4.952<br>7.54E-08	3.244<br>1.39E-05		
Sparc			2.979<br>1.85E-06			
Lox	-2.023<br>9.3E-03		2.164<br>5.41E-05	2.345<br>4.11E-05		

Table S4: List of differentially expressed related to the mitochondrial activity along with their FC and P-value (white background: downregulated and gray background: upregulated)

Gene	D3	D7	D10	D14	D21	D28
Gpx1	-2.95<br>7.9E-06	-3.164<br>8.9E-06				
Prdx2	-3.235<br>1.80E-06	-3.29<br>1.96E-06				
Prdx3	-2.429<br>6.92E-07	-2.396<br>6.30E-06				
Oxct1	-3.157<br>2.92E-05	-3.299<br>4.41E-05				
Slc25a11	-2.06872<br>2.56E-03	-2.1049<br>7.53E-05				
Slc25a20		-2.10498<br>2.86E-06				
Bckdk	-2.633<br>1.03E-05	-2.198<br>7.8E-04				
Tmem14c	-2.46345<br>1.07E-05	-2.075<br>1.5E-04				
Atp5b	-2.64177<br>1.15 E-07	-2.618<br>3.93E-07				
Atp5f1	-2.5096<br>2.22E-06	-2.491<br>5.78E-04				
Tomm7	-2.322<br>1.25E-06	-2.221<br>2.95E-06				
Cs	-2.588<br>8.93E-05	-2.573<br>5.75E-05				
Fh1	-2.907<br>6.72E-06	-2.907<br>2.90E-06				
Suclg1	-2.205<br>1.87E-06	-2.16<br>1.97E-05				
Uqcrc2	-2.277<br>7.33E-06	-2.278<br>6.90E-06				

Cyc1	-3.796<br>1.75E-06	-3.73<br>3.08E-06			
Coq9	-3.328<br>1.06E-05	-3.539<br>2.28E-07			
Cyp27a1	-2.164<br>5.91E-06	-2.303<br>2.05E-06			
Hspa9	-2.499<br>1.1E-03	-2.468<br>2.1E-04			
Isca1	-2.204<br>1.39E-06	-2.368<br>3.89E-06			
Csde1	-3.414<br>3.15E-05	-3.501<br>1.19E-04			
Pdk4	-2.35<br>2.17E-05	-2.255<br>1.43E-03			
Etfa	-3.692<br>8.17E-05	-3.623<br>1.36E-05			
Gstp1	-2.566<br>5.11E-05	-2.7103<br>1.76E-05			
Timm8b	-3.668<br>2.37E-05	-3.866<br>6.14E-06			
Vdac1		-2.549<br>3.77E-06			
Vdac3	-2.609<br>7.90E-04	-2.732<br>9.26E-06			-2.045<br>7.20E-03
Cpt1b			-2.445<br>1.03E-05	-2.156<br>3.40E-07	
Cox8b			-2.514<br>1.53E-06	-2.289<br>3.19E-05	
Cox7a1			-2.516<br>1.76E-07	-2.491<br>1.83E-05	
Cox6a2			-2.436<br>1.86E-07		
Ckmt2	-3.476<br>3.33E-07	-3.454<br>7.87E-07	-2.395<br>4.44E-07	-2.112<br>7.73E-06	

Sdhc				-2.1253<br>6.59E-07		
Lyrm5				-2.1653<br>1.15E-05		
Dusp26				-2.0079<br>4.45E-04		
Slc25a37					-2.4586<br>1.42E-05	-2.684<br>8.88E-06
Slc25a5			2.109133<br>3.81E-06			

Table S5: List of differentially expressed related to the ribosomal activity along with their FC and P-value (white background: downregulated and gray background: upregulated)

Gene	D3	D7	D10	D14	D21	D28
Rps6	-3.925<br>3.40E-05	-3.542<br>7.50E-04				
Rpl24	-3.0135<br>5.92E-05	-2.987<br>3.45E-05				
Rpl27	-2.704<br>2.35E-07	-2.7882<br>5.13E-06				
Rpl31	-4.360<br>5.82E-06	-3.889<br>1.54E-05				
Rps2	-4.3006<br>5.25E-05	-4.174<br>1.72E-07				
Rpl36al	-2.352<br>1.55E-06	-2.328<br>2.62E-05				
Ubb	-3.837<br>5.82E-08	-3.823<br>3.13E-06				
Mrpl53	-2.1221<br>3.84E-06	-2.8511<br>1.10E-03				
Mrpl33	-2.035<br>1.41E-05					
Npm1	-2.5708<br>1.12E-05	-2.758<br>5.11E-05				

Metap1		-2.02905<br>1.13E-03				
E2f2					-2.040<br>9.61E-05	-2.350<br>2.00E-03
Hnrnph1	-2.261<br>1.92E-06	-2.319<br>1.49E-05			-2.053<br>2.82E-06	-2.003<br>1.20E-04
Mt3			2.1711<br>2.90E-03	2.177<br>3.19E-03	2.2716<br>2.54E-03	
Pdia3			2.083<br>3.82E-06			
